# An Observational Review of Tonsillectomy and Appendectomy Procedures Conducted at a Tertiary Care Hospital

**DOI:** 10.7759/cureus.79708

**Published:** 2025-02-26

**Authors:** Mohammed Habiel, Fatima Alharmoodi, Khadija Almaghribi, Manayer Alteneiji, Mohammed Alblooshi, Mahdi Al-Taher

**Affiliations:** 1 Department of General Surgery, Tawam Hospital, Al Ain, ARE; 2 Department of General Surgery, College of Medicine and Health Sciences, Al Ain, ARE; 3 Department of Pediatric Surgery, Tawam Hospital, Al Ain, ARE

**Keywords:** appendectomy, retrospective study, surgical epidemiology, tertiary hospital, tonsillectomy

## Abstract

Background: Tonsillectomy and appendectomy are the most frequently performed surgical procedures in pediatric and adult populations. However, comprehensive data examining their occurrence within the same hospital setting remain sparse. This study aimed to characterize the demographic and clinical profiles of patients undergoing tonsillectomy and assess the frequency and outcomes of those who additionally underwent appendectomy.

Methods: A retrospective review was performed on 337 patients who underwent tonsillectomy at a tertiary care hospital from 2015 to 2017. Data collected included age, nationality, sex, year of tonsillectomy, and details of any concurrent or subsequent appendectomy, such as complicating factors (e.g., perforation and abscess), imaging findings (appendicolith), and pathology results. Statistical analyses were performed using Minitab 18 (Minitab, Inc., State College, PA).

Results: Among 337 patients, five (1.5%) underwent appendectomy in addition to tonsillectomy. Overall, 78.3% (264/337) of the tonsillectomy group were pediatric vs. 21.7% (73/337) adult, while 60% (3/5) of the appendectomy group were pediatric and 40% (2/5) adult (p = 0.33). The mean (standard deviation) age across the entire cohort was 16.17 (10.48) years, and 55.2% (186/337) were men. No cases of complicated appendicitis were identified among the appendectomy patients, although three (representing 0.9% of the total cohort and 60% of the appendectomy subgroup) exhibited an appendicolith on imaging. Pathology findings confirmed acute appendicitis in four (1.2%) of these cases and a normal appendix in one (0.3%). Statistical comparisons revealed no significant difference in median age between patients undergoing tonsillectomy alone and those who also had an appendectomy (p = 0.86), nor in distribution by gender across procedures (p = 0.78).

Conclusion: This review highlights the young demographic profile of patients undergoing tonsillectomy in a tertiary care hospital and underscores the relatively low incidence of appendectomy in this cohort. Despite the small subset of appendectomy cases, timely intervention may have contributed to the absence of complicated appendicitis. Notably, institutionwide data suggest that negative appendectomies remain rare overall, indicating a need for further research into this phenomenon. These findings underscore the need for continued surveillance and future multicenter studies to better elucidate potential shared risk factors and outcomes associated with these commonly performed surgical procedures.

## Introduction

Tonsillectomy and appendectomy remain among the most frequently performed surgical procedures in pediatric and adult populations, reflecting their continued importance in managing common otolaryngological and gastrointestinal conditions, respectively [1,2]. Tonsillectomy, typically indicated for recurrent tonsillitis or obstructive sleep apnea, has garnered increasing interest regarding its complication rates, indications, and outcomes in diverse age groups [3]. Meanwhile, appendectomy remains the gold-standard treatment for acute appendicitis, particularly in complicated cases, such as those involving perforation or abscess [4]. Despite the vast literature on each procedure independently, there remains a relative paucity of data describing the co-occurrence or demographic overlap of these procedures within a single tertiary care hospital setting, particularly when examining both pediatric and adult cohorts.

Recent publications emphasize the value of retrospective reviews for identifying patterns in healthcare delivery, especially in smaller populations or unique clinical settings [5]. Retrospective studies provide a rich historical context that can reveal fluctuations in surgical indications, patient demographics, and postoperative outcomes [5,6]. Moreover, methodological considerations in retrospective surgical studies are critical for ensuring data reliability [7]. Filling this gap is essential for enhancing patient care and guiding future research that explores the relationships or trends between these two procedures.

Accordingly, the objective of this study was to conduct an observational review of all adult and pediatric patients who underwent tonsillectomy in a tertiary care hospital over a defined period from 2015 to 2017 while recording the frequency and outcomes of those who likewise required appendectomy. Recent multicenter analyses have underscored the value of large database reviews in pediatric surgery [8]. By correlating patient demographics, surgical indications, and histopathology findings, we aimed to ascertain trends and inform decision-making processes regarding preoperative evaluation, surgical planning, and postoperative care. Given the unique co-occurrence of cases undergoing both tonsillectomy and appendectomy, we sought to identify any emergent themes or noteworthy factors linking these procedures within our patient population.

To achieve this, we conducted a retrospective chart review encompassing all patients who underwent tonsillectomy (and those with documented appendectomy) within the hospital between 2015 and 2017. Data variables included age, nationality, sex, year of surgery (both tonsillectomy and appendectomy), complicated appendicitis status (perforated or with abscess), presence of an appendicolith, and pathology results. By analyzing these parameters, we aimed to contribute to the body of evidence on surgical epidemiology within the context of a tertiary healthcare facility and to outline key demographic and clinical patterns that may guide future research and practice.

## Materials and methods

This retrospective observational study was conducted at Tawam Hospital, a tertiary care facility in Al Ain, United Arab Emirates. Electronic medical records housed in the Cerner system (Cerner Corporation, Kansas City, MO) were reviewed for all patients who underwent tonsillectomy between January 2015 and December 2017. Data were initially gathered for all age groups. The variables extracted included demographic information (age, nationality, and sex), year of tonsillectomy, appendectomy status (yes/no), year of appendectomy, indicators of complicated appendix (perforation or abscess), radiological findings (appendicolith), and final pathology results.

Inclusion criteria required patients to have undergone a documented tonsillectomy during the specified study period, with complete demographic and surgical information available in the records. Any cases where key data elements were missing were excluded. Data were systematically coded and secured in a password-protected database to ensure confidentiality. Descriptive statistics (mean, standard deviation, SD, frequency, and percentage) were used to characterize the study population, providing an overview of the demographic and clinical variables. Statistical analyses were performed using Minitab 18 (Minitab, Inc., State College, PA). All patient information was anonymized prior to analysis in adherence to the ethical and privacy regulations of the hospital.

Decision to proceed to surgery

In our institution, the decision to perform an appendectomy is generally based on a combination of clinical presentation (e.g., right lower quadrant pain, guarding, and rebound tenderness), laboratory findings (particularly elevated inflammatory markers such as leukocyte count and C-reactive protein), and imaging studies (ultrasound or computed tomography). Although no single diagnostic scoring system was formally adopted for all cases during the study period, the Alvarado score was often referenced as part of the clinical workflow to support the diagnosis of acute appendicitis. Specifically, in the five cases that ultimately underwent appendectomy, the patients initially presented with classic symptoms of acute appendicitis, which were corroborated by elevated inflammatory markers and imaging findings (e.g., presence of an appendicolith). These convergent data points, rather than any single factor, determined the decision to proceed with surgical intervention.

## Results

Demographic characteristics

A total of 337 patients were included in this retrospective review (Table 1). The mean (SD) age of the cohort was 16.17 (10.48) years. Men comprised 186 (55.2%) and women 151 (44.8%) of the sample. Among the various nationalities represented, Emirati patients constituted the largest group at 271 (80.4%). Other nationalities included Yemeni (3.0%), Omani (2.7%), Egyptian (2.1%), Jordanian (1.8%), and smaller proportions from multiple additional countries.

**Table 1:** Demographic summary SD: standard deviation

Variables	Results, n = 337
Age in years, mean (SD)	16.17 (10.48)
Nationality, n (%)	
Emirati	271 (80.4%)
Yemeni	10 (3%)
Omani	9 (2.7%)
Egyptian	7 (2.1%)
Jordanian	6 (1.8%)
Indian	5 (1.5%)
Saudi	4 (1.2%)
Sudanese	4 (1.2%)
Filipino	3 (0.9%)
Palestinian	3 (0.9%)
Syrian	2 (0.6%)
Bangladeshi	2 (0.6%)
Kuwaiti	2 (0.6%)
Pakistani	2 (0.6%)
Mauritanian	1 (0.3%)
American	1 (0.3%)
British	1 (0.3%)
Finnish	1 (0.3%)
Pitcairn Islander	1 (0.3%)
Somali	1 (0.3%)
Iranian	1 (0.3%)
Sex, n (%)	
Male	186 (55.2%)
Female	151 (44.8%)
Tonsillectomy year, n (%)	
2017	128 (38%)
2015	124 (36.8%)
2016	85 (25.2%)
Tonsillectomy, n (%)	
No	0 (0%)
Yes	337 (100%)
Appendectomy, n (%)	
No	332 (98.5%)
Yes	5 (1.5%)

Tonsillectomy and appendectomy overview

Across the three-year period (2015-2017), 128 (38.0%) patients underwent tonsillectomy in 2017, 124 (36.8%) in 2015, and 85 (25.2%) in 2016 (Table 1). Of the 337 patients, five (1.5%) also underwent appendectomy, whereas the remaining 332 (98.5%) did not. Advanced details regarding these five appendectomy cases are listed in Table 2; no patient had complicated appendicitis (0%), although three (0.89%) had radiographic evidence of an appendicolith. Pathology findings for the appendectomy specimens ranged from acute appendicitis with serositis (0.59%) to normal-appearing appendix (0.3%) (Table 2).

**Table 2:** Advanced details of patients undergone appendectomy

Complicated appendix	Results, n (%)
Yes	0 (0%)
No	5 (1.48%)
Did images show appendicolith?	
No	2 (0.59%)
Yes	3 (0.89%)
Pathology result	
Acute appendicitis with serositis	2 (0.59%)
Acute transmural appendicitis	1 (0.3%)
Normal appearance	1 (0.3%)
No evidence of significant inflammation, no evidence of malignancy	1 (0.3%)

Timing of appendectomy relative to tonsillectomy

Table 3 illustrates the timing of appendectomy relative to tonsillectomy. One patient (0.3%) underwent appendectomy before tonsillectomy, while four (1.19%) underwent appendectomy after tonsillectomy.

**Table 3:** Timing of appendectomy relative to tonsillectomy

Period of appendectomy occurrence	Number of patients (%)
Appendectomy before tonsillectomy	1 (0.3%)
Appendectomy after tonsillectomy	4 (1.19%)

Among the five patients who underwent an appendectomy in addition to tonsillectomy, four (80%) underwent laparoscopic appendectomy, while one (20%) had an open procedure. This detail underscores our institutional preference for minimally invasive techniques.

Age distribution analysis

**Figure 1:**
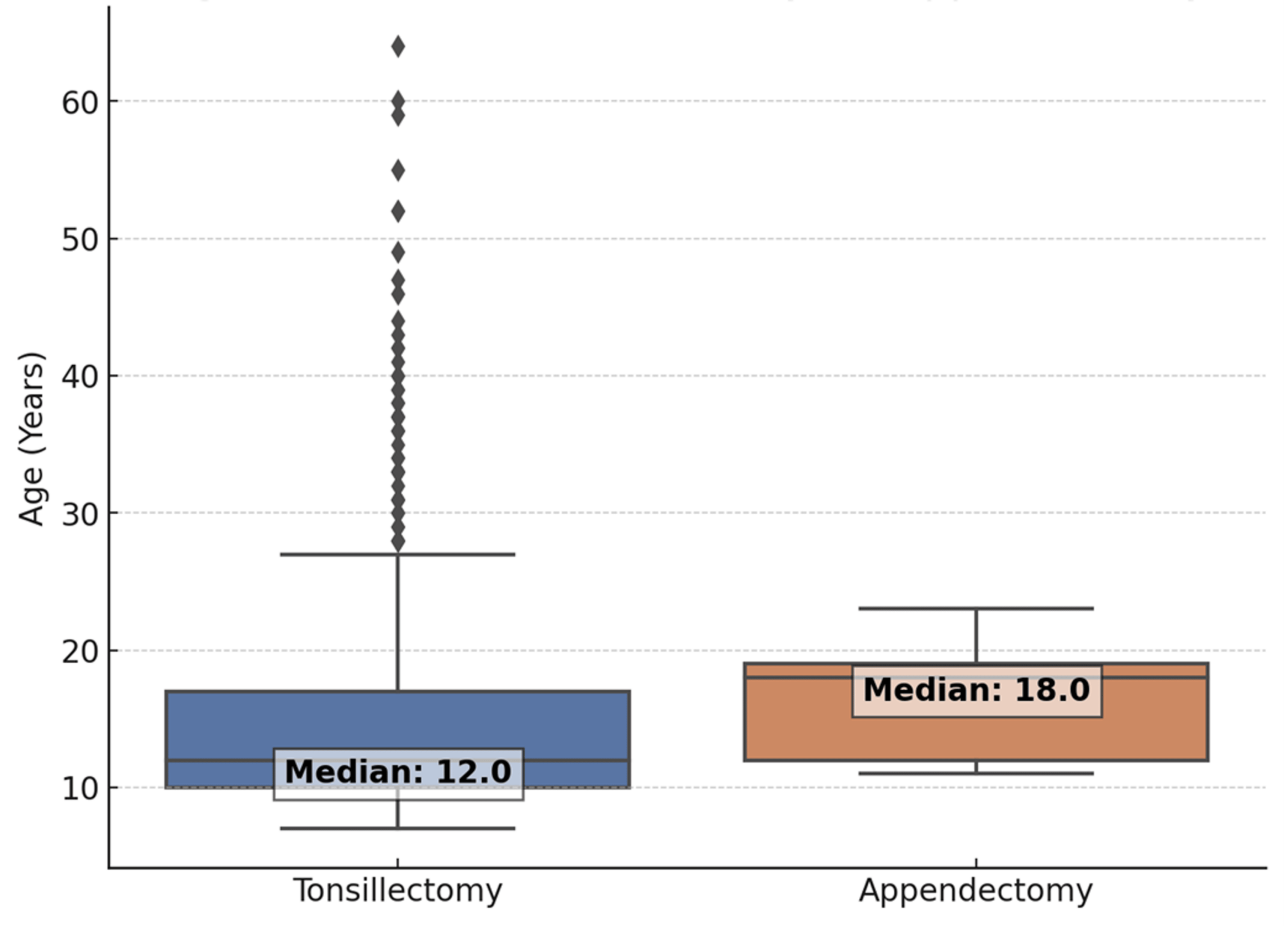
Age distribution comparison between tonsillectomy and appendectomy patients

**Figure 2:**
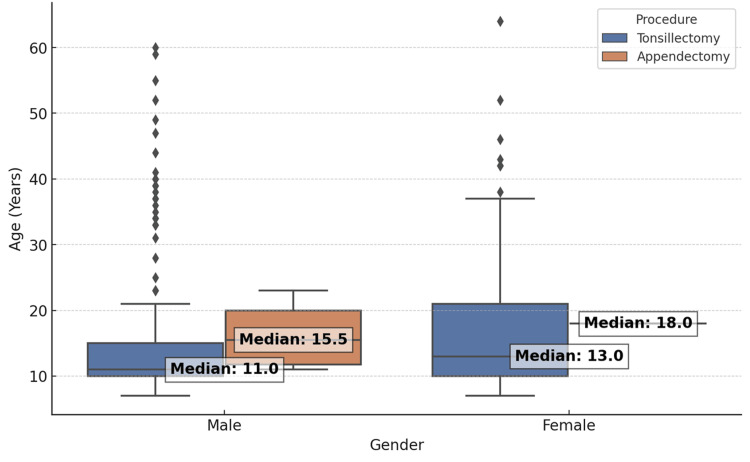
Age distribution categorized by gender and procedure

Figure 1 compares the age distribution between those who underwent tonsillectomy alone and those who also had an appendectomy (p = 0.86). Figure 2 displays the age distribution stratified by gender and procedure (p = 0.78). No statistically significant differences in median age were observed between these groups.

**Figure 3:**
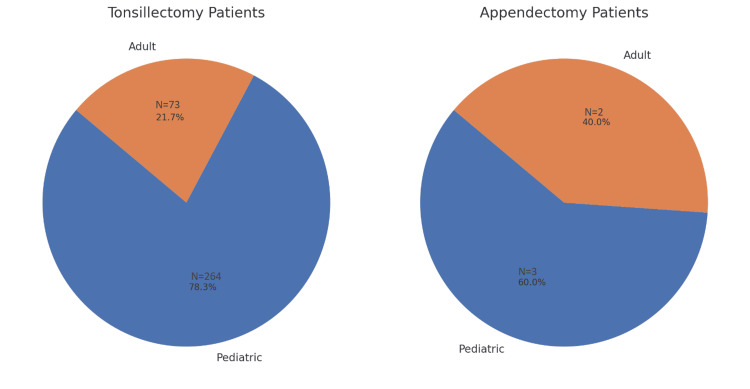
Comparison of age categories among patients undergoing tonsillectomy and appendectomy

Figure 3 illustrates the relative proportions of pediatric vs. adult patients who underwent tonsillectomy and appendectomy.

Among the 337 tonsillectomy patients, 78.3% were pediatric, and 21.7% were adults. By contrast, among the five patients who underwent appendectomy, 60% were pediatric, and 40% were adult. Although there is an apparent difference in the proportion of pediatric patients between these two groups, a chi-square test revealed that this difference did not reach statistical significance (p = 0.33), likely reflecting the small sample size in the appendectomy cohort.

## Discussion

The findings of this retrospective analysis highlight several noteworthy observations regarding tonsillectomy and appendectomy in a tertiary care hospital setting. First, the predominance of younger patients undergoing tonsillectomy is consistent with existing literature, which identifies tonsillectomy as a commonly performed procedure in pediatric populations due to chronic tonsillitis and obstructive sleep apnea [3,9]. In addition, a systematic review reported similar complication rates and risk factors in pediatric tonsillectomy [10]. Although a subset of older patients was also observed, the overall age distribution confirms that tonsillectomy is primarily indicated among children and adolescents [5]. Our data additionally showed a small proportion of patients who experienced the co-occurrence of tonsillectomy and appendectomy over the study period, a finding that aligns with trends observed in larger population-based studies on appendectomy outcomes [11].

An interesting point of note is the absence of complicated appendicitis (i.e., perforation or abscess) among the five patients who underwent appendectomy. This observation may signal robust preoperative diagnostic pathways, allowing for timely surgical intervention before complications develop [4]. Moreover, a systematic review highlighted that the presence of an appendicolith can predict complicated appendicitis, although this was not observed in our cohort [12]. However, the small number of appendectomy patients limits our ability to draw more definitive conclusions about the true incidence of complicated appendicitis within this hospital’s population. Additionally, three of the five appendectomy cases showed radiographic evidence of an appendicolith, emphasizing the utility of imaging in guiding early intervention and potentially averting more severe disease [13].

Intra-abdominal abscess (IAA) formation is a recognized postoperative complication, particularly following appendectomy for complicated appendicitis. A recent retrospective study by Mulita et al. compared IAA formation after laparoscopic and open appendectomy for both complicated and uncomplicated appendicitis, finding no significant difference between the two approaches (with IAA incidences of 5.19% for laparoscopic and 7.07% for open procedures in complicated cases) [14]. Although our series did not include any cases of complicated appendicitis or subsequent IAA formation, these findings underscore the importance of both early intervention and meticulous surgical technique in minimizing postoperative complications. Further studies with larger sample sizes are warranted to elucidate the impact of surgical approach on IAA risk.

From a demographic standpoint, the sample included predominantly Emirati patients, mirroring the broader population profile of the hospital’s catchment area. The presence of multiple other nationalities, though in small numbers, highlights the institution’s culturally diverse patient base. Such diversity, albeit limited, can serve as a springboard for future studies examining how cultural, genetic, or environmental factors influence the incidence or outcomes of surgical conditions such as chronic tonsillitis and acute appendicitis [1]. Moreover, because only five appendectomy cases were identified within the broader cohort, these results reinforce the specialized focus on otolaryngological surgeries at this facility.

Although this study did not identify statistically significant differences in age distributions between those who had a tonsillectomy alone and those who additionally had an appendectomy, it does underscore the potential for overlapping patient populations. It remains an open question whether this overlap is purely incidental or reflective of shared predisposing factors (e.g., general susceptibility to infection or local healthcare utilization trends). Future multicenter investigations with larger sample sizes could help clarify potential associations or risk profiles. In addition, longer term follow-up might ascertain whether prior tonsillectomy influences the management or outcome of subsequent appendicitis and vice versa [2].

In summary, this study’s results illustrate clear age-related patterns in tonsillectomy, a minimal overlap of appendectomy cases, and effective early identification of appendicitis within the sampled tertiary hospital. While the restricted sample size for appendectomy patients calls for caution in extrapolating broader conclusions, the data offer valuable baseline insights into local surgical demographics and outcomes. Future research could further explore potential shared determinants of tonsillectomy and appendectomy, evaluate strategies for improved perioperative care, and expand our understanding of pediatric and adult surgery trends in similar tertiary care settings.

## Conclusions

This retrospective review offers a focused view of tonsillectomy and appendectomy patterns in a tertiary hospital over a three-year period. The data showed that most tonsillectomy patients were children or adolescents, with a small yet clinically relevant fraction also undergoing appendectomy. Although only five patients underwent appendectomies, the absence of complicated cases in this subgroup indicates prompt recognition and intervention for acute appendicitis. However, this approach led to one histologically normal appendix, resulting in a 20% negative appendectomy rate within this small cohort. Notably, institutionwide data show that negative appendectomies are uncommon in general, pointing toward additional study of this phenomenon.

These findings provide preliminary evidence regarding the demographic profile and procedural overlap in this single-institution experience. Further, larger scale multicenter investigations would enhance our understanding of co-occurrence rates, risk factors, and long-term outcomes. Ultimately, our study underscores the importance of ongoing surveillance to monitor trends in surgical outcomes and the implementation of robust surgical protocols to ensure timely intervention and optimal care, thereby minimizing complications such as perforation or abscess.
